# Role of High-Resolution CT Thorax in Diagnosing Interstitial Lung Disease and Its Association With Smoking and Connective Tissue Disorder

**DOI:** 10.7759/cureus.31107

**Published:** 2022-11-04

**Authors:** Jainam A Doshi, Krati S Mundhra, Dharita S Shah, Sahil N Shah, Tamanna V Patel, Anand Bhatt

**Affiliations:** 1 Radiology, Sardar Vallabhbhai Patel Institute of Medical Sciences and Research, Ahmedabad, IND

**Keywords:** thoracic complication of rheumatoid arthritis, usual interstitial pneumonia, non-specific interstitial pneumonia, idiopathic interstitial pneumonias, smoking and interstitial lung disease, interstitial lung disease, pulmonary arterial hypertension, connective tissue disease associated interstitial lung disease

## Abstract

Introduction

Interstitial lung diseases (ILDs) primarily affect the interstitium, an alveolar wall tissue between the capillary endothelium and the alveolar epithelium. The term ‘interstitial,’ however, is misleading since alveolar spaces, peripheral airways, and vessels can be involved in most of these disorders.They often require a multidisciplinary diagnosis i.e., an integration of clinical, radiological, and pathological findings. A chest radiograph is relatively insensitive because of nonspecific patterns.

Generally, these disorders can progress to irreversible pulmonary fibrosis and are an important cause of morbidity and mortality. It is critical to make a prompt and accurate diagnosis of the underlying causes so that patients can be managed appropriately. ILD is subdivided into idiopathic interstitial pneumonia, of which idiopathic pulmonary fibrosis (IPF) is one subset, and diffuse parenchymal lung diseases, which may be secondary to a variety of occupational or environmental exposures or others. They can complicate multiple rheumatic or connective tissue diseases (CTDs). Apart from ILD, other forms of lung damage involving the pleura, vasculature, airways, and lymphatic tissue can complicate CTDs.

Aims

Aims include studying the role of high-resolution computed tomography (HRCT) in diagnosing various ILDs based on morphologic patterns, evaluating the correlation between ILD and various connective tissue disorders and the prevalence of complications in such patients, and evaluating the association of smoking with various ILDs.

Methods

This is a retrospective study in which HRCT thorax was performed on a 128-slice Philips CT scanner machine on 50 patients from December 2020 to February 2022 in SVP Hospital, Ahmedabad. No age or gender bias was followed.

Result

Out of 50 patients studied, 19 (38%) patients had the usual interstitial pneumonia (UIP) pattern and 12 (24%) had the nonspecific interstitial pneumonia (NSIP) pattern. These two were the most common among all ILD patterns. Other patterns found were hypersensitivity pneumonitis (5; 10%), respiratory bronchiolitis-related ILD (3;6%), and organizing pneumonia (2; 4%). In nine patients, the morphologic pattern was either subtle (3; 6%) or mixed (6; 12%), and the final diagnosis remained inconclusive; patients were advised clinical correlation and biopsy. Eleven (22%) patients had a history of smoking. Among smokers, the most common pattern was UIP while all patients with respiratory bronchiolitis (RB) ILD had a history of smoking. Fourteen (28%) patients showed a positive association with CTD. Among them, rheumatoid arthritis (RA) was the most common CTD and the most common pattern among RA patients was UIP. Ten (20%) of patients developed pulmonary arterial hypertension, of which two patients who had connective tissue disorder developed pulmonary arterial hypertension at a young age (24 years). The rest of the patients who developed pulmonary arterial hypertension were above 45 years of age. Among these, two were smokers.

Conclusion

HRCT plays an important role in the diagnosis of ILD on the basis of various morphological patterns. CTD plays a significant role in the development of ILD. UIP is the most common ILD among patients with a smoking history and RA. NSIP Is the most common in patients with CTD other than RA. Pulmonary arterial hypertension (PAH) develops early in patients with CTD. There is a significant risk of the development of PAH in patients with chronic ILD.

## Introduction

Interstitial lung diseases (ILDs) primarily affect the interstitium, a tissue of the alveolar wall between the capillary endothelium and the alveolar epithelium. The term ‘interstitial’ is misleading because alveolar spaces, peripheral airways, and vessels can be involved in most of these disorders. Clinical presentations include dyspnea, cough, clubbing of fingernails, and weight loss. Pulmonary function tests show a restrictive pattern. They often require a multidisciplinary diagnosis, i.e., an integration of clinical, radiological, and pathological findings. However, after the introduction of high-resolution computed tomography (HRCT), typical CT-based morphologic patterns associated with idiopathic interstitial pneumonia (IIP) could be precisely obtained. Therefore, radiologists play an important and critical role in its diagnosis and characterization [1].

A chest radiograph is relatively insensitive but remains the primary radiological tool for screening clinically suspected patients. Many diseases remain occult or are not correctly diagnosed on chest X-ray because of a nonspecific ‘reticulonodular pattern.’ Almost 10-20% of the patients with histologically proven interstitial lung disease have a normal CXR. HRCT is highly sensitive in detecting interstitial lung diseases, despite its sensitivity not being 100%. The specificity for the characterization of different ILDs has been documented and appears to be better than conventional radiography [2-4].

In general, these disorders can progress to irreversible pulmonary fibrosis and are an important cause of morbidity and mortality among all age groups. It is thus important that a prompt and accurate diagnosis of the underlying cause is made to ensure that the patients can be given appropriate management. ILD is subdivided into idiopathic interstitial pneumonia, of which idiopathic pulmonary fibrosis (IPF) is one subset, and diffuse parenchymal lung diseases, which may be secondary to a variety of occupational or environmental exposures, or as discussed in the present review. They can complicate multiple rheumatic or connective tissue diseases (CTDs). These diseases include systemic sclerosis. where ILD occurs in most patients, and rheumatoid arthritis (RA), polymyositis/dermatomyositis (PM/DM), Sjogren's syndrome, systemic lupus erythematosus (SLE), undifferentiated CTD, and mixed CTD, where ILD is a less frequent complication. In addition to ILD, other forms of lung damage involving the pleura, vasculature, airways, and lymphatic tissues can complicate CTDs. This review will cover only one complication, i.e., pulmonary arterial hypertension [4-6].

## Materials and methods

Type of study

This is a retrospective study. Clinically suspected ILD patients who were referred for HRCT by the general medicine and pulmonary medicine departments to the department of radiodiagnosis at the tertiary care Sardar Vallabhbhai Patel (SVP) Hospital, Smt. Nathiba Hargovandas Lakhmichand (NHL) Municipal Medical College (MMC), Ahmedabad, were included in this study. The data were collected retrospectively from the picture archiving and communication system (PACS) and hospital information system (HIS). Over a year, between December 2020 and February 2022, a total of 50 cases were studied.

Duration of the study

The study took place from December 2020 to February 2022.

Study site

The study was conducted at the Department of Radiodiagnosis, Sardar Vallabhbhai Patel Institute of Medical Sciences and Research (SVPIMSR), Smt. NHL Municipal Medical College; Ahmedabad.

Inclusion criteria 

The study included all age groups irrespective of their sex and all patients with the interstitial lung disease-like pattern on HRCT.

Imaging parameters

All scans were performed on a 128-slice Philips CT scanner (Amsterdam, Netherlands) with the following scan parameters: slice thickness of 1.00 mm; collimation of 128 x 1.00; pitch of 0.95; 160 mAs; 120 kVp

Exclusion criteria

The study excluded patients with associated lung pathology like consolidation, mass, or occupational lung disease and patients developing interstitial lung disease post-coronavirus disease 2019 (COVID-19) infection.

Methodology

CT scans were performed with a Philips Ingenuity 128-slice CT machine with the above-mentioned imaging parameters. Plain CT scans studies are included in the present study. No contrast-enhanced scans were included in the study. For selected patients, inspiratory and expiratory films were obtained. Volumetric data of HRCT were reconstructed in multiple planes in high-resolution lung windows and soft tissue windows. Patients were diagnosed based on clinical histories like occupation, exposures, allergy, family history, addiction, course of the disease, and the various HRCT patterns, including reticulation, traction bronchiectasis, honeycombing, ground glass opacities, nodules, and emphysematous changes. Biopsy findings are not included in this article [7].

## Results

Our study indicates that out of the 50 patients, most show patterns of reticulation and traction bronchiectasis, followed by ground-glass opacities and honeycombing. Other less common findings were emphysematous changes and centrilobular nodules. Of the 50 patients studied, 19 (38%) had the usual interstitial pneumonia (UIP) pattern and 12 (24%) had the nonspecific interstitial pneumonia (NSIP) pattern. These two were the most common among all ILD patterns. Other patterns found were hypersensitivity pneumonitis (5, 10%), respiratory bronchiolitis-related ILD (3, 6%), and organizing pneumonia (2, 4%). In nine patients, the morphologic pattern was either subtle (3, 6%) or mixed (6, 12%) and their final diagnosis remained inconclusive; therefore, they were advised clinical correlation and biopsy [8].

Among the study population, 28 patients were male and 22 patients were female. The clinical symptoms were progressive dyspnea, which was the most common (44, 88%), cough (37, 74%), fever (27, 54%), and weight loss (15, 30).

Table 1 shows the age-wise distribution of patients in the study.

**Table 1:** Age-wise distribution

Age group(years)	Number of patients	Percentage
1-10	1	2
11-20	1	2
21-30	4	8
31-40	0	0
41-50	2	4
51-60	14	28
61-70	17	34
71-80	3	6
80-81	8	16

Table 2 lists the HRCT patterns observed in various ILDs. 

**Table 2:** HRCT patterns of various interstitial lung disease HRCT: high-resolution computed tomography

HRCT Pattern	Reticulation	Traction bronchiectasis	Ground-glass opacities	Honeycombing	Nodules	Emphysematous changes
Usual interstitial pneumonia	19	19	8	19	0	5
Non-specified interstitial pneumonia	12	8	12	6	0	0
Hypersensitivity pneumonitis	0	1	5	0	5	1
Respiratory bronchiolitis-Interstitial lung disease	3	3	3	0	0	3
Organizing pneumonia	0	0	2	0	0	0
Early interstitial lung disease	2	0	6	0	0	1
Inconclusive	6	4	5	3	4	1

Table 3 lists the distribution of diseases among the study population. 

**Table 3:** Distribution of disease among the study population

Pattern	Number of patients	Percentage
Usual interstitial pneumonia	19	38
Non-specified interstitial pneumonia	12	24
Hypersensitivity pneumonitis	5	10
Respiratory bronchiolitis-Interstitial lung disease	3	6
Organizing pneumonia	2	4
Early interstitial lung disease	3	6
Inconclusive	6	12

Connective tissue disease

Among 50 patients, 14 were found positive for one of the following: rheumatoid arthritis (most common, 9), systemic lupus erythematosus (1), dermatomyositis (2), mixed connective tissue disorder (1), or Sjogren's disease(1). Among rheumatoid arthritis-positive patients, the most common pattern was usual interstitial pneumonia (6) while the NSIP pattern in most common in the other five cases [9-10].

Smokers and Non-smokers

Among 50 patients, 11 were smokers. Among smokers, the most common pattern was usual interstitial pneumonia. Patients with the respiratory bronchiolitis (RB) ILD pattern on HRCT had a 100% association with smoking. All three patients had a history of smoking [11-12].

Patients Who Developed Pulmonary Arterial Hypertension

Among 50 patients, 10 patients were diagnosed with pulmonary arterial hypertension. Two patients 24 years of age who had a connective tissue disorder developed pulmonary arterial hypertension. The rest of the patients who developed pulmonary arterial hypertension were above 45 years of age. Among these, two patients had a history of smoking [6,13].

**Figure 1:**
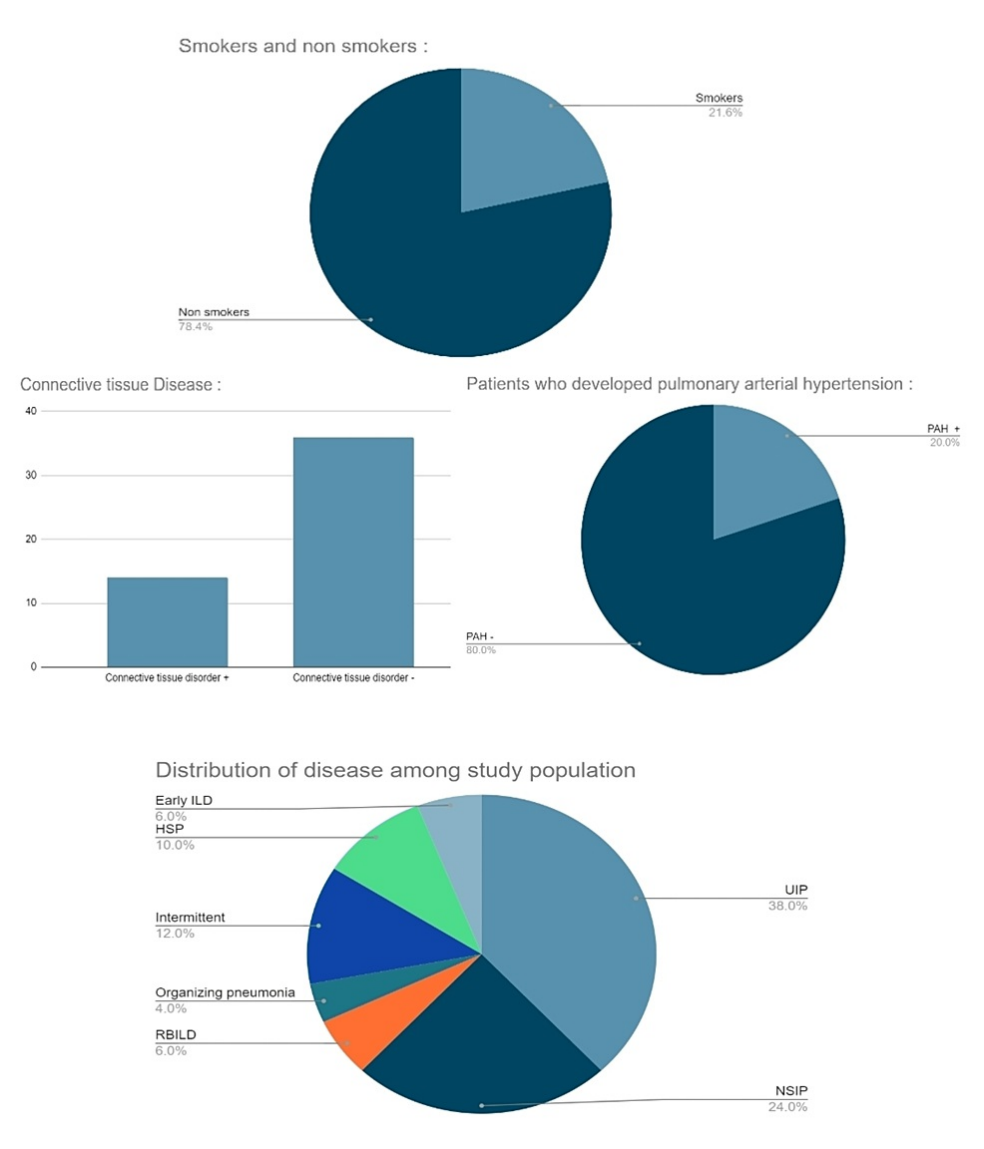
Graphical representation of various results

Figure 1 is a graphical representation of the above-mentioned results.

.

## Discussion

Interstitial lung disease is fundamentally related to inflammation and fibrosis of the lung interstitium and its contents with resultant changes in the lung parenchyma. Idiopathic interstitial pneumonia was classified according to histological patterns, clinical features, and radiological patterns as described below.

UIP is the most common of the IIPs. NSIP is the next most frequent, followed by cryptogenic organizing pneumonia (COP), desquamative interstitial pneumonia (DIP), RB ILD, and acute interstitial pneumonia (AIP), which are less common, while LIP and PPFE are rare. All the IIPs give restrictive patterns on pulmonary function tests. UIP can be further classified as per various morphologic patterns on HRCT. The diagnostic criteria are described below.

ILDs are very often found associated with collagen vascular diseases. The most prevalent collagen vascular disease among ILD patients is rheumatoid arthritis while systemic sclerosis is CTD in which the majority of patients develop ILD.

The age group of the subjects ranged from 7 to 89 years and most (34.0%) were recorded in the age group of 61-70 years. In the present study of 40 patients, 28 (56%) were males and 22 (44%) were female.

Table 4 describes the various typical morphological patterns of different ILDs [14].

**Table 4:** The American Thoracic Society-European Respiratory Society classification of IIPs (revised in 2013) IIP: idiopathic interstitial pneumonitis, IPF: Idiopathic pulmonary fibrosis, UIP: usual interstitial pneumonitis, NSIP: non-specific interstitial pneumonitis, RB ILD: respiratory bronchiolitis-related interstitial lung disease

IIP group and clinical -radiologic-Pathologic Diagnosis	CT pattern	Typical CT distribution	Typical CT findings	CT Differential Diagnosis
Chronic fibrosing IIPs				
IPF	Usual interstitial pneumonia	Peripheral, subpleural, basal	Reticular opacities, honeycombing, traction bronchiectasis or bronchiectasis, architectural distortion, focal ground-glass attenuation	Collagen vascular disease, hypersensitivity pneumonitis, asbestosis, sarcoidosis
Idiopathic NSIP	NSIP	Peripheral basal, symmetric	Ground-glass attenuation, irregular lines, traction bronchiectasis, consolidation	UIP, desquamative interstitial pneumonia, Cryptogenic organizing pneumonia, hypersensitivity pneumonitis
Smoking-related IIPs				
Desquamative interstitial pneumonia.	Desquamative interstitial pneumonia	Lower zone, peripheral predominance in most cases	Ground glass attenuation, reticular lines, cysts	RB ILD, NSIP, hypersensitivity pneumonitis
RB-ILD	Respiratory bronchiolitis	Often upper lung predominant, centrilobular	Bronchial wall thickening, centrilobular nodules, patchy ground glass opacity	Desquamative interstitial pneumonia, NSIP, hypersensitivity pneumonitis
Acute or subacute IIPs				
Cryptogenic organizing pneumonia	Organizing pneumonia	Subpleural or peribronchial	Patchy consolidation or nodular, perilobular pattern, reverse halo sign	Infection, aspiration, Eosinophilic pneumonia, NSIP, vasculitis, sarcoidosis, mucinous adenocarcinoma, lymphoma
Acute interstitial pneumonia	Diffuse alveolar damage	Diffuse or patchy	Consolidation and ground glass opacity, often with lobular sparing, traction bronchiectasis	Hydrostatic edema, pneumonia, pulmonary hemorrhage, acute eosinophilic pneumonia
Rare IIPs				
Lymphoid interstitial pneumonia	Lymphoid interstitial pneumonia	More commonly, lower lung predominant	Centrilobular nodules, ground glass attenuation, septal and bronchovascular thickening, thin-walled cysts	NSIP, sarcoidosis, Langerhans cell histiocytosis, and other cystic lung diseases
Idiopathic pleuroparenchymal fibroelastosis	Idiopathic pleuroparenchymal fibroelastosis	Peripheral, upper lung predominant	Pleural thickening and subpleural fibrotic changes	Sarcoidosis, pneumoconiosis, familial pulmonary fibrosis, connective tissue disease, hypersensitivity pneumonitis

The findings of the present study are consistent with a study conducted by Coultas DB et al. [15]. In this study, out of a total of 50 patients, 19 (38.0%) showed the HRCT pattern reflecting UIP, 12 (24%) showed NSIP, five (10%) had hypersensitivity pneumonitis (HSP), and two (4%) had COP while only three (6%) showed changes of RB ILD. Six (12%) patients showed HRCT patterns with an inconclusive diagnosis. DIP, AIP, and lymphocytic interstitial pneumonia (LIP) were not seen in any of the 50 patients [8]. These findings correlate well with studies done by Coultas DB et al. and Oliveira, Daniel Simões et al. [14,15].

**Figure 2:**
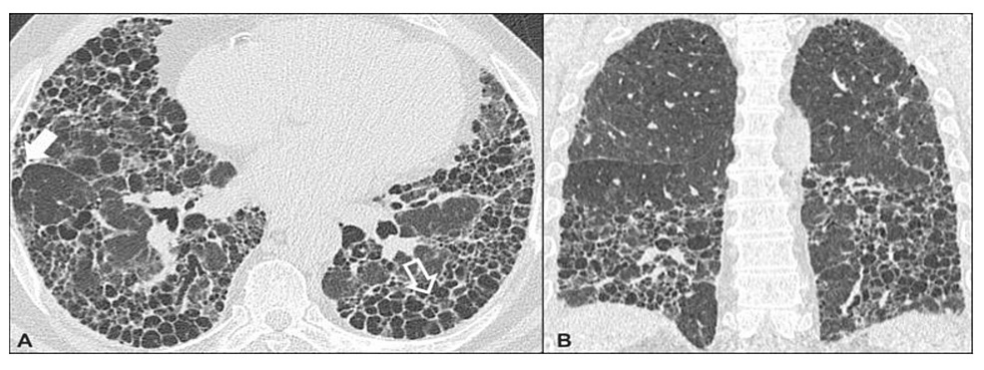
Usual interstitial pneumonia A) Axial image at lower lobes level; B) Coronal image shows honeycombing (hollow white arrow) and interstitial septal thickening( solid white arrow)

**Figure 3:**
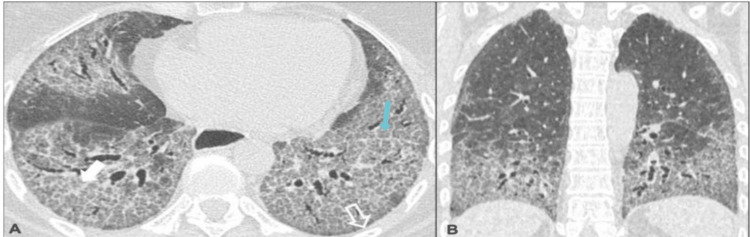
Nonspecific interstitial pneumonia A) Axial section; B) Coronal section images show ground-glass opacities (solid white arrow), traction bronchiectasis, and reticulation (light blue arrow)

**Figure 4:**
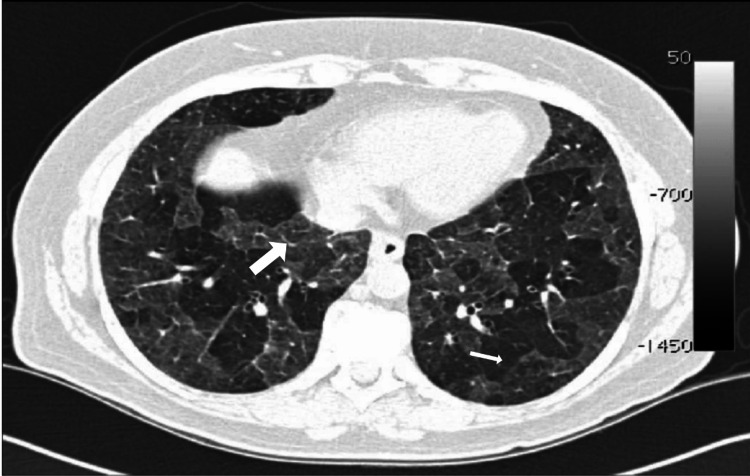
Hypersensitivity pneumonitis Axial image with lung window shows areas of ground-glass opacities (thick white arrow) with normal spared areas of lung (thin white arrow)

**Figure 5:**
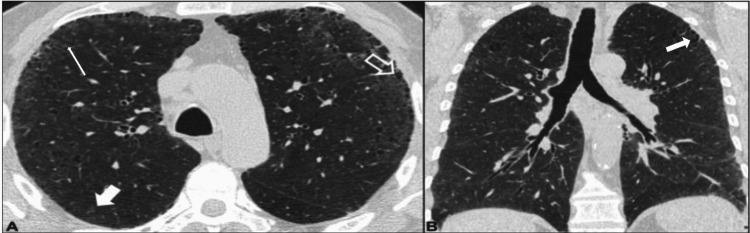
Respiratory bronchiolitis-associated interstitial lung disease A) Axial section; B) Coronal section of the thorax in the lung window shows centrilobular (thick white arrow) and para-septal (hollow white arrow) emphysematous changes and reticulation (thin white arrow)

**Figure 6:**
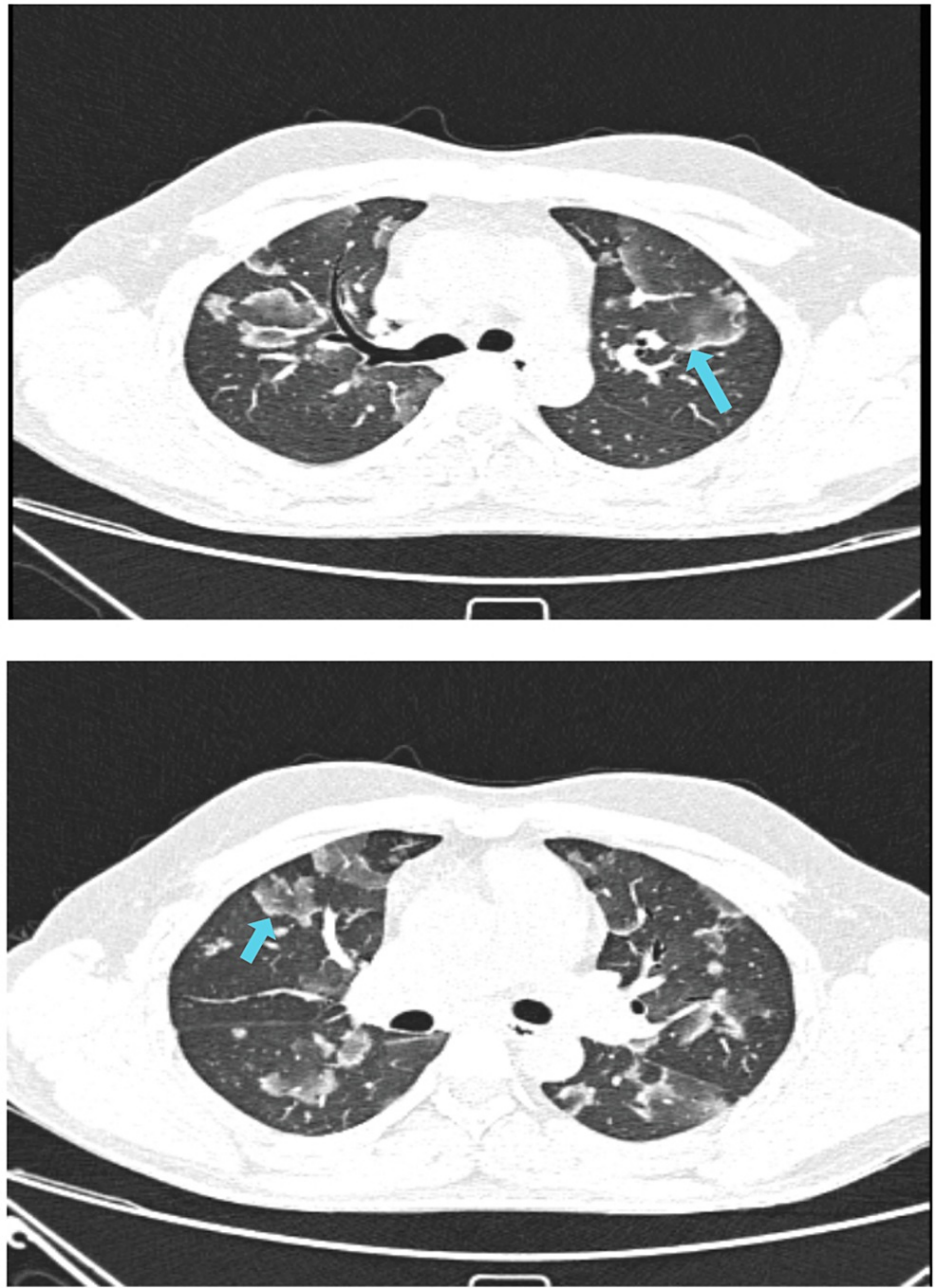
Cryptogenic organizing pneumonia Axial sections of the thorax at different levels show ground-glass opacities with a surrounding rim of consolidation giving a reverse halo or atoll sign

Figures 2-6 demonstrate various typical morphological patterns of the different ILDs described in Table 2.

Among smokers, the most common pattern was usual interstitial pneumonia. Patients with the RB ILD pattern on HRCT had a 100% association with smoking. RB ILD and desquamative interstitial pneumonia have been named as smoking-related interstitial lung diseases [11-12].

Among 50 patients, 10 were diagnosed with pulmonary arterial hypertension. Two patients who were 24 years old had a connective tissue disorder and developed pulmonary arterial hypertension. The rest of the patients who developed pulmonary arterial hypertension were above the age of 45. Among these, two patients were smokers. From past studies, it is known that CTD is the second most common cause of PAH. We conclude that patients diagnosed with connective tissue disorder have a high chance of developing complications such as PAH at a young age as compared to the general population. While in general, chronicity of the disease is responsible for the development of PAH [13].

## Conclusions

HRCT plays an important role in the diagnosis of interstitial lung disease on the basis of various morphological patterns. There is a significant role of CTD in the development of ILD.

UIP is the most common ILD among patients with a smoking history and rheumatoid arthritis. NSIP is the most common pattern in patients with CTD other than rheumatoid arthritis. There is an early development of PAH in patients with CTD. There is also a significant risk of the development of PAH in patients with a chronic history of ILD.
